# Baseline preferences for oral pre-exposure prophylaxis (PrEP) or dapivirine intravaginal ring for HIV prevention among adolescent girls and young women in South Africa, Uganda and Zimbabwe (MTN-034/IPM-045 study)

**DOI:** 10.1371/journal.pone.0287525

**Published:** 2023-06-23

**Authors:** Kenneth Ngure, Barbara A. Friedland, Daniel W. Szydlo, Sarah T. Roberts, Morgan Garcia, Lisa Levy, Carolyne A. Akello, Krishnaveni Reddy, Thesla Palanee-Phillips, Pippa Macdonald, Bekezela Siziba, Lydia Soto-Torres, Sybil Hosek, Sharon L. Hillier, Gonasagrie Nair, Connie Celum, Ariane van der Straten

**Affiliations:** 1 School of Public Health, Jomo Kenyatta University of Agriculture and Technology, Nairobi, Kenya; 2 Department of Global Health, University of Washington, Seattle, WA, United States of America; 3 Population Council, Center for Biomedical Research, New York, NY, United States of America; 4 Statistical Center for HIV/AIDS Research and Prevention, Fred Hutchinson Cancer Research Center, Seattle, WA, United States of America; 5 Women’s Global Health Imperative (WGHI), RTI International, Berkeley, California, United States of America; 6 FHI 360, Durham, North Carolina, United States of America; 7 Johns Hopkins University Research Collaboration, Makerere University, Kampala, Uganda; 8 Wits Reproductive Health and HIV Institute, Faculty of Health Sciences, University of the Witwatersrand, Johannesburg, South Africa; 9 University of Zimbabwe Clinical Trials Research Centre, Harare, Zimbabwe; 10 National Institute of Allergy and Infectious Diseases, National Institutes of Health, Bethesda, Maryland, United States of America; 11 Department of Psychiatry, John Stroger Hospital of Cook County, Chicago, Illinois, United States of America; 12 Department of Obstetrics, Gynecology, and Reproductive Sciences, University of Pittsburgh, Pittsburgh, Pennsylvania, United States of America; 13 Centre for Medical Ethics and Law, Stellenbosch University, Stellenbosch, South Africa; 14 Department of Medicine, University of Washington, Seattle, Washington, United States of America; 15 Department of Epidemiology University of Washington, Seattle, Washington, United States of America; 16 Center for AIDS Prevention Studies, Dept of Medicine, University of California, San Francisco, San Francisco, California, United States of America; 17 ASTRA Consulting, Kensington, CA, United States of America; University of the Witwatersrand, SOUTH AFRICA

## Abstract

**Introduction:**

Adolescent girls and young women (AGYW) in sub-Saharan Africa are disproportionately affected by the HIV epidemic and face an array of challenges using proven behavioral and biomedical prevention methods. To address the urgent need for expanding prevention options, we evaluated the baseline preferences of HIV prevention methods among participants enrolled in the MTN-034/REACH crossover trial along with their stated product preference prior to product initiation.

**Methods:**

AGYW aged 16–21 years were enrolled at 4 study sites: Cape Town and Johannesburg, South Africa; Kampala, Uganda; and Harare, Zimbabwe and randomly assigned to the sequence of using oral PrEP and the dapivirine ring for 6 months each, followed by a choice period in which they could choose either product (or neither) for an additional six months. Eligible AGYW were HIV-negative, not pregnant and using effective contraception for at least two months prior to enrollment. Descriptive statistics were used to summarize demographic and behavioral data while multinomial analysis was used to determine predictors of stated product preference (ring or oral PrEP).

**Results:**

Of the 247 AGYW enrolled in REACH, 34% were aged 16–17 and 89% had a primary partner.The median age of sexual debut was 16 years and 40% had ever been pregnant. At screening, 35% of participants were diagnosed with a sexually transmitted infection (STI), 39% had an AUDIT-C score associated with harmful drinking and 11% reported intimate partner violence in the past 6 months. Overall, 28% of participants, had CESD-10 scores suggestive of depressive symptoms (≥12) in the past week. At baseline, similar proportions stated a preference for the ring and oral PrEP (38.1% and 40.5% respectively), with 19% of participants stating they preferred both products equally. Only study site was significantly associated with product preference (P<0.05) with AGYW from Johannesburg having higher odds of preferring the ring and those from Kampala having higher odds of preferring both options equally.

**Conclusions:**

We successfully enrolled African AGYW with a clear unmet need for HIV prevention. The balanced preference between the two products suggests that multiple biomedical prevention options may be appealing to this age group and could address their prevention needs.

## Introduction

In sub-Saharan Africa, adolescent girls, and young women (AGYW) aged 15–24 years old are disproportionately affected by the HIV epidemic, with the majority of new infections in 15–19-year-olds occurring among females [1]. HIV continues to be the leading cause of death among 10–19-year-olds in Africa and the second most common cause of death among adolescents globally [2]. An array of factors contribute to AGYW’s HIV risk, including lack of awareness regarding safe sexual practices, inability to negotiate male condom use, biological development, and lack of psychosexual maturation [3, 4]. These factors impede AGYW’s use of proven behavioral and biomedical HIV prevention methods and highlight the urgent need to expand options available to young African women.

Truvada® (emtricitabine [FTC] and tenofovir disoproxil fumarate [TDF]) used once daily for oral pre-exposure prophylaxis (PrEP) has been a cornerstone of HIV prevention efforts since its approval by the US Food and Drug Administration (FDA) in 2012 [5]. The World Health Organization (WHO) recommended offering oral PrEP to select key populations at high risk of HIV acquisition in 2014 [6], and subsequently expanded their recommendations for broader provision based on evidence that itis safe, cost-effective, and highly efficacious in reducing HIV acquisition regardless of age, gender, or mode of transmission [7, 8]. However, challenges with daily pill use and low persistence, especially among AGYW [9, 10] indicate that some young women who could benefit from daily oral PrEP do not choose to or are unable to use it consistently.

A one-month intravaginal ring containing the antiretroviral drug dapivirine (ring) was shown to reduce the risk of HIV-1 acquisition by approximately 30% in two Phase 3 trials among healthy female adults in sub-Saharan Africa [11, 12]. The ring was recently approved by the European Medicines Agency (EMA) and has been recommended by the WHO as a prevention option for women who are unwilling or unable to use oral PrEP [13, 14]. In post-hoc analyses for both studies, greater risk reduction (up to 75%) was observed among participants with higher ring adherence [15]. The ring is a promising alternative to oral PrEP; however, data are needed to determine if the ring can successfully address challenges to oral PrEP adherence. Furthermore, there is a need for more data on how 16–17 year-old AGYW in sub-Saharan Africa will use the ring, since it has not yet been studied in that population.

To that end, the Microbicide Trials Network (MTN) implemented MTN-034/REACH (Reversing the Epidemic in Africa with Choices in HIV Prevention)to evaluate the safety, adherence, acceptability, and preference of the ring versus oral PrEP. Here we report on the baseline characteristics and pre-use product preferences of participants enrolled in the trial

## Methods

### Study design

**Fig 1:**
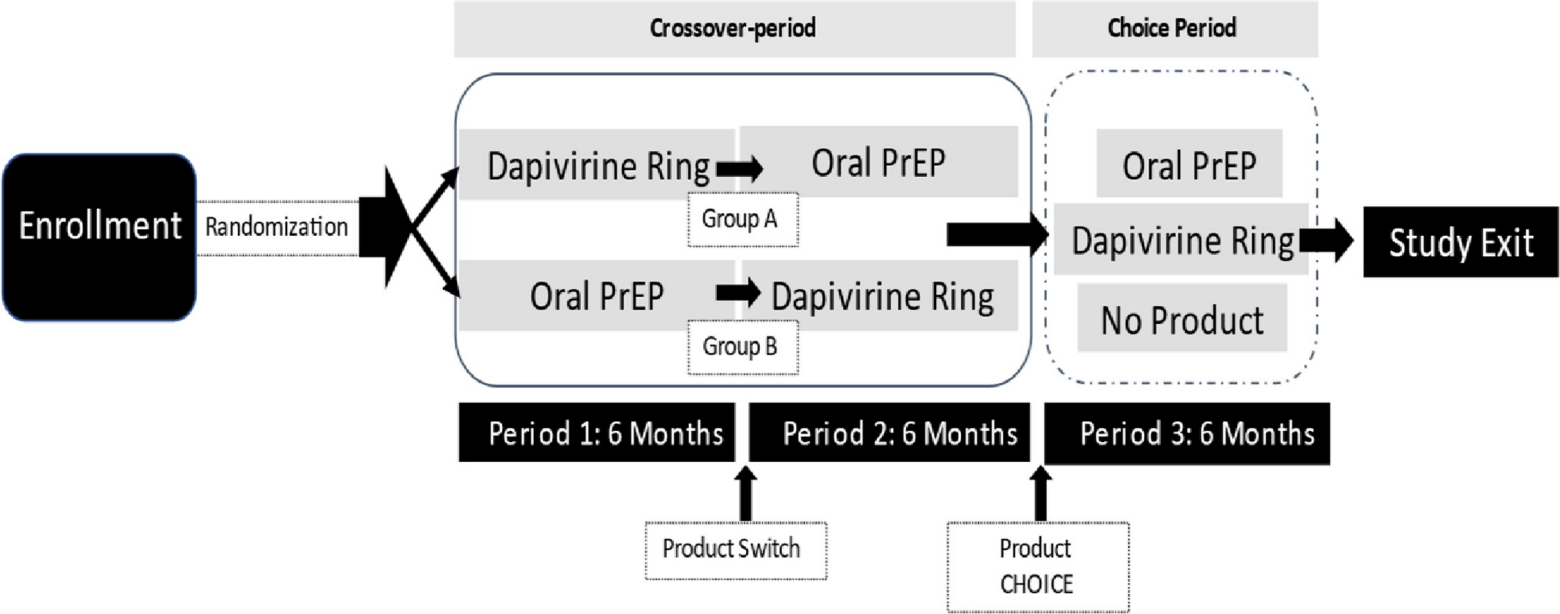
REACH study design and visit schedule.

REACH was a multi-site, two-arm, randomized, open-label, crossover Phase 2a trial (ClinicalTrials.gov: NCT03593655) that aimed to enroll ˜300 participants across four sites in Africa (Kampala, Uganda; Harare, Zimbabwe; and Cape Town and Johannesburg, South Africa). Participants were randomized (1:1) to one of two sequences of product use: using the ring for 24 weeks and then switching to oral PrEP for a second 24 weeks or using oral PrEP for 24 weeks and then switching to the ring (Fig 1). After completing the crossover period, participants could choose oral PrEP, the ring, or neither to use in 24-week Choice period.

### Study population and baseline testing prior to enrollment

Eligible participants were 16–21 years of age, HIV-negative per rapid test at screening and enrollment, not pregnant at screening or enrollment, using effective contraception for at least two months prior to enrollment (for those not on contraceptive at screening, the study offered effective contraception at screening, encouraging long-acting contraceptives), and healthy based on physical exam, pelvic exam and laboratory testing. Pregnancy and breastfeeding were exclusionary because the ring had not yet been studied in in an African population of this age. There were no other criteria related to sexual behavior or HIV vulnerability.

### Recruitment

Participants were recruited from a variety of settings including, but not limited to, adolescent and primary care health clinics, family planning clinics, HIV testing facilities, gynecology clinics, institutions of higher learning, community-based youth centers, schools, and other community locations. Community education strategies, including use of pelvic models to demonstrate ring insertion, an educational video, and other printed materials were shared individually and in group sessions and via social media platforms to inform potential participants and parents/guardians about the study. Although the study was designed to enroll approximately 300 participants, screening and enrollment on all MTN studies was paused on March 28, 2020, due to the COVID-19 pandemic and its impact on research operations. Because the study had reached 82% of its accrual target, the study was not reopened for screening and enrollment. Therefore, a total of 247 were enrolled in the study. It was a requirement that at least a third of enrolled participants were aged 16–17 to provide important safety data in this age group. In Uganda, because recruitment of minors from schools was prohibited by the local IRB, emancipated minors from the community, including school dropouts, were targeted. All recruitment materials were approved by sites’ IRBs/Ethics Committees (ECs) prior to use.

### Measures

Mental health conditions, especially depression and alcohol abuse, have been shown to predict PrEP adherence in multiple studies [16]. To explore the connection between mental health and adherence in this population, we assessed participants’ mental health at baseline using several scales administered through audio computer-assisted self-interviewing (ACASI). Participants who reported mental-health related challenges were counseled by study staff or referred to community organizations offering mental health, substance use, gender-based violence, and other services. We used the 10-item Center for Epidemiological Studies–Depression scale (CES-D-10) a short self-report questionnaire to screen for depressive symptoms in the past week [17]. Options for each item, such as “I feel lonely,” ranged from 0 (rarely or none of the time) to 3 (all the time). Total scores range from 0 to 30, with higher scores suggestive of a greater severity of depressive symptoms. Although Andersen determined that a score of 10 was an optimal cut-off for individuals at risk of depression [17], a study validating the CES-D-10 in South Africa determined that slightly higher cut-offs ranging (11,12, and 13 for Afrikaans, Zulu and Xhosa respectively) were more predictive of depressive symptoms in this population [18], leading us to use a score of > = 12 to be indicative of depressive symptoms in our study population.

Alcohol use disorders were assessed using the Alcohol Use Disorders Identification Test (AUDIT-C), a 3-item questionnaire (range 0–12). Higher scores are indicative of more harmful alcohol practices [19]. In women, a score of ≥3 is indicative of an alcohol abuse disorder.

We used an abbreviated version Brief Self-Control Scale (BSCS) [20] to measure level of perceived self-control to explore associations between self-control and adherence. Each of the seven items in the scale, such as “I am good at resisting temptation” are scored from 1 (not at all) to 5 (very much). Total scores range from 7 to 35, with higher scores indicative of higher levels of self-control.

Social support was assessed using the Multidimensional Scale of Perceived Social Support (MSPSS) [21], which has been validated in Uganda [22] and South Africa [23]. Each of the four items in the peer sub-scale (such as “My friends really try to help me”) is scored on a 5-point Likert scale from 1 (Strongly Disagree) to 5 (Strongly Agree). The overall score ranges from 1 to 20, with higher scores indicative of greater peer social support.

Participants were tested for C*hlamydia trachomatis* (CT) and *Neisseria gonorrhoeae* (GC) by nucleic acid amplification testing, *Trichomonas vaginalis* (TV) by rapid test, and syphilis by rapid plasma reagin followed by a treponemal-specific confirmatory assay. AGYW with positive STI test results received treatment and were eligible to enroll once symptoms resolved to ensure they had no genital inflammation before starting ring use.

### Data collection and analysis

Baseline demographics and clinical data were collected via interviewer-administered case report forms (CRFs) at both the screening and enrollment visits, while baseline behavioral data were self-reported by participants via audio computer-assisted self-interviewing (ACASI) at enrollment. Medians were used to summarize the mental health scales. We explored significant associations (p < 0.05) between demographic and behavioral characteristics and participants’ baseline product preferences for oral PrEP, the ring, or both products equally using multinomial logistic regression models, with “preference for oral PrEP” as the reference group. The model for site was adjusted for age (16–17-year-olds vs 18–21-year-olds), conversely, the model for age was adjusted for site, and all other models were adjusted for age and site. Analyses were carried out in SAS Version 9.4 (SAS Institute, Cary, NC, USA).

### Ethics statement

The study was approved by site Institutional Review Boards/Ethics Committees (IRBs/ECs) prior to use: University of Cape Town Faculty of Health Sciences Human Research Ethics Committee, University of Witwatersrand Human Research Ethics Committee, University of Zimbabwe Joint Research Ethics Committee, Johns Hopkins Medicine Institutional Review Board and the Joint Clinical AIDS Research Center IRB of Uganda. Written informed consent was obtained from adult participants, and from minors who were legally able to consent as per local regulations. Written informed assent and parental/guardian consent was obtained from all other minors, and those who turned 18 during the study subsequently signed the adult informed consent form (ICF). In addition to ICFs, the REACH Protocol Team worked with study staff and community representatives to develop appropriate materials about the study and a standardized approach to implementing the informed assent/consent process at all study sites.

## Results

Between February 2019 and March 2020, 247 AGYW were enrolled in REACH. Participants were median age of 18 years (interquartile range [IQR] 17,19) and most (87%) were unmarried, however other background characteristics varied substantially by site (such as self-earned income, who participants lived with, and alcohol use). About one-third (37%) of participants were currently attending either secondary or tertiary schools. The highest level of education attained by participants was secondary school and above (86%), except in Kampala, where approximately one-third of participants had attended secondary school and about half had only attended primary school. The percentage of participants who earned their own income varied considerably by site, from 3% in Cape Town to 52% in Kampala(Table 1).

**Table 1:** Baseline socio-demographic characteristics of AGYW enrolled in the REACH study (n = 247).

Characteristic	SA ‐ Cape Town	SA–Johannesburg	Uganda–Kampala	Zimbabwe–Harare	All Sites
**Participants Enrolled**	60	67	60	60	247
**Age (Median [IQR])**	18 (17,19)	18 (17,19)	18 (17,19)	18 (17,19)	18 (17,19)
Minors (16–17)	18(30.0%)	28(47.0%)	19(28.0%)	20(33.0%)	86(34.0%)
**Marital Status**					
Single	60 (100.0%)	65(97.0%)	53(88.0%)	36(60.0%)	214(87.0%)
Married/Cohabiting	0(0%)	2(3.0%)	7(12.0%)	21(35.0%)	30(12.0%)
Separated or divorced	0(0%)	0(0%)	0(0%)	3(5%)	3(1%)
**Currently attending school**	33(55.0%)	36(54.0%)	9(15.0%)	14(23.0%)	92(37.0%)
**Highest Level of School Attended**					
Primary	1(2.0%)	0(0.0%)	28(47.0%)	4(7.0%)	33(13.0%)
Secondary	55(92.0%)	57(85.0%)	22(37.0%)	55(93.0%)	189(77.0%)
Higher (e.g., college or university)	4(7.0%)	10(15.0%)	9(15.0%)	0(0.0%)	23(9.0%)
**Earns own income**	2(3.0%)	6(9.0%)	31(52.0%)	14(23.0%)	53(21.0%)
**Has a regular place to store her things**	43(71.7%)	60(89.6%)	52(86.7%)	48(80.0%)	203(82.2%)
**Who participant lives with**					
Alone	0.0	1(2.0%)	6(11.0%)	2(4.0%)	9(4.0%)
Parents	47 (78.0%)	48 (75.0%)	29 (54.0%)	32 (70.0%)	156 (70.0%)
Lives with non-parent family members	6 (10.0%)	10 (16.0%)	16 (30.0%)	8 (17.0%)	40 (18.0%)
Lives with non-family members	7(12.0%)	5(8.0%)	3(6.0%)	4(9.0%)	19(8.0%)
**Worry about not getting enough food in the last 30 days**					
Never	26(43.3%)	26(38.8%)	31(51.7%)	32(53.3%)	115(46.6%)
Rarely + sometimes	30(50.0%)	33(49.3%)	21(35.0%)	33(38.3%)	107(43.3%)
Often (more than 10 times)	4(6.7%)	8(11.9%)	8(13.3%)	5(8.3%)	25(10.1%)
**Median age of sexual debut (Min, Max)**	16.0 (13, 18)	16.0 (13,19)	15.0 (12,18)	16.0 (13,18)	16.0 (12,19)
**Median age of first use of contraception (min, Max)**	16.0 (13,19)	17.0 (14,21)	17.5 (15,20)	17.0 (15,20)	17.0 (13,21)
**Contraceptive Method at Baseline**					
Implants	11(18.3%)	30(44.8%)	37(61.7%)	33(55.0%)	111(44.9%)
Copper IUD	1(1.7%)	8(11.9%) ^a^	9(15.0%)	9(15.0%)	27(10.9%)
Injectable contraceptive	48(80.0%)	29 (43.3%)	14(23.3%)	18(30.0%)	109(44.1%)
**Ever Pregnant (self-report)**	5(8.0%)	21(31.0%)	32(53.0%)	41(68.0%)	99(40.0%)
**Caregiver for** **>****1 child**	9(15.0%)	22(33.0%)	21(35.0%)	34(57.0%)	86(35.0%)
**Diagnosed with STI**	28(47.0%)	25(37.0%)	19(32.0%)	15(25.0%)	87(35.0%)
**Worry about HIV infection in the Next Year**					
Very Worried	22(37.0%)	19(28.0%)	6(10.0%)	24(40.0%)	71(29.0%)
Somewhat worried	7(12.0%)	8(12.0%)	0(0.0%)	14(23.0%)	29(12.0%)
A little worried	23(38.0%)	23(34.0%)	10(17.0%)	12(20.0%)	68(28.0%)
Not at all worried	8(13.0%)	17(25.0%)	44(73.0%)	10(17.0%)	79(32.0%)
**CES-D-10 Depression Scale** (Median, IQR)	7.0 (5,10)	8.5 (4,13)	9.5 (6,15)	6.0 (5,11)	7.0 (5,12)
**Depressive symptoms (CESD> = 12)**	9(15.0%)	22^b^ (33.0%)	24(40.0%)	11^c^ ^(^23.0%)	66 ^d^ (28.0%)
**Positive (AUDIT-C ≥ 3) indicative of hazardous drinking/alcohol abuse disorder**	38(63.0%)	31(46.0%)	13(22.0%)	15^e^ (25.0%)	97^f^ (39.0%)
**New Brief Self-Control Scale** (median, IQR)	27 (24,30)	27 (25,31)	24.5 (22,29)	28 (23,29)	27 (23,30)
**Peer Social Support (MSPSS)** (Median, IQR)	15 (12,16)	16 (12,16)	14 (12,16)	14.5 (11,16)	15 (12,16)

^a^ Combination of the “copper IUD” (n = 26) and “hormonal IUD” (n = 1)

^b^ Denominator = 66

^c^ Denominator = 47

^d^ Denominator = 233

^e^Denominator = 59

^f^Denominator = 246

The median age of sexual debut was 16 (IQR 12,19) which was one year younger than the median age of first contraception use, 17 (IQR 13, 21). By self-report, 40% of participants had previously been pregnant, ranging from 8% in Cape Town to 68% in Harare. By contrast, STI diagnosis at baseline was most prevalent among Cape Town based participants (47%) compared to the other sites (25%-37%).

Overall, 28% of participants, and 40% of participants from Kampala, had CESD-10 scores suggestive of depressive symptoms (≥12) in the past week. Nearly 40% of participants overall, and 63% of participants from Cape Town had a positive score on the AUDIT-C, suggesting a high prevalence of alcohol use disorders in the study cohort. In contrast, moderate levels of self-control (median score of 27 out of 35) and peer social support (median score of 15 out of 20) were reported across sites.

### Behavioral characteristics

As shown in Table 2, 89.1% of participants reported having a primary sexual partner. Median age of primary partners was 23 years (IQR 20,25). Approximately 60% of participants had been in their relationship for more than one year and received financial support from their primary partners. The majority of participants reported that their primary partners were HIV-negative (73.6%), while 25% did not know his status and only three women had a partner who they reported was living with HIV. A minority reported that their primary partners were definitely (7.7%) or possibly (15.5%) having sex with other partners. Many participants had experienced verbal (30%) or physical (15%) abuse or forced sex (11%) by an intimate partner, with reports varying substantially by site.

**Table 2:** Partner/partnership characteristics.

Characteristic	SA ‐ Cape Town	SA–Johannesburg	Uganda–Kampala	Zimbabwe–Harare	All Sites
**Participants Enrolled**	60	67	60	60	247
**Currently has a primary sexual partner**	53(88.3%)	61(91.0%)	54(90.0%)	52(86.7%)	220(89.1%)
**Median age of primary sexual partner (IQR)**	21 (19,23)	22 (22,24)	23 (20,26)	24 (22,26)	23 (20,25)
**Duration of partnership with primary partner**					
Less than 3 months	5(9.4%)	3(4.9%)	4(7.4%)	5(9.6%)	17(7.7%)
3–12 months	22(41.5%)	14(23.0%)	18(33.3%)	14(26.9%)	68(30.9%)
More than 12 months	26(49.1%)	44(72.1%)	32(59.3%)	32(61.5%)	134(60.9%)
Skipped by participant	0 (0%)	0(0%)	0(0%)	1(1.9%)	1(0.5%)
**Primary sex partner provides financial and/or material support**	18(34.0%)	32(52.5%)	44(81.5%)	42(80.8%)	136(61.8%)
**HIV status of primary sex partner**					
HIV positive	1(1.9%)	1(1.6%)	0	1(1.9%)	3(1.4%)
HIV negative	34(64.2%)	47(77.0%)	43(79.6%)	38(73.1%)	162(73.6%)
Don’t know	18(34.0%)	13(21.3%)	11(20.4%)	13(25.0%)	55(25.0%)
**Primary sex partner had sex with another partner besides the study participant in the last 3 months**					
Knows so	4(7.5%)	4(6.6%)	6(11.1%)	3(5.8%)	17(7.7%)
Thinks so	6(11.3%)	10(16.4%)	8(14.8%)	10(19.2%)	34(15.5%)
**Physical abuse from intimate partner, last 6 months**	3(5.0%)	3(4.5%)	11(18.3%)	19(31.7%)	36(14.6%)
**Verbal abuse from intimate partner, last 6 months**	8(13.3%)	19(28.4%)	20(33.3%)	28(46.7%)	75(30.4%)
**Forced sex from intimate partner, last 6 months**	0(0%)	4(6.0%)	14(23.3%)	9(15.0%)	27(10.9%)
**Forced Sex from non-intimate partner, last 6 months.**	1(1.7%)	5(7.5%)	9(15.0%)	2(3.3%)	17(6.9%)
**Vaginal sex, past 3 months**	50 (83.3%)	64(95.5%)	49(81.7%)	40(69.0%)	203*(82.9%)
**Vaginal Sex in the past 30 days**					
N	50	64	49	40	203
Median (IQR)	4(2,5)	3.5 (2,7)	5 (2,8)	4 (1,11)	4 (2,7)
**Condom used last vaginal sexual act**	31(62.0%)	37(57.8%)	24(49.0%)	21(50.0%)	113(55.1%)
**Anal sex in the last 3 months**	6(10.0%)	4(6.0%)	6 (10.0%)	3(5.0%)	19(7.7%)
**Condom used last act of anal sex**	5(83.3%)	3(75.0%)	5(83.3%)	3(100.0%)	16(84.2%)
**Number of male sexual partners in the past 3 months (categorical)**					
0	5(8.3%)	0(0%)	3(5.0%)	4(6.7%)	12(4.9%) *
1	36(60.0%)	44(65.7%)	27(45.0%)	39(65.0%)	146(59.1%)
2+	19(31.7%)	23(34.3%)	30(50.0%)	17(28.3%)	89(36.0%)
**Median number of sexual partners in the past 3 months (IQR)**	1(1,2)	1(1,2)	1(1,3)	1(1,2)	1(1,2)
**Transactional sex in the past 6 months**	7(11.7%)	10(14.9%)	33(55.0%)	16(26.7%)	66(26.7%)

*The numbers are mismatched due to inconsistency in participants’ self-report (If 12 participants had 0 partners, then the total number of participants who had vaginal sex in the last 3 months should be 235)

Overall, 82.2% of participants reported having had sex at least once in the three months preceding screening. Among these participants, vaginal sex occurred four times per month on average (IQR 2,7). Condom use at last sex was reported by a little more than half of participants, with minimal variation by site. Although only 19 participants reported having anal sex in the 3 months prior to screening, 84% of those participants reported having used a condom during the last anal sex act. The majority of participants reported having had only one partner in the three months before screening, whereas 36% reported multiple partners. More than one-quarter of the participants reported engaging in transactional sex, which varied by site from 12% in Cape Town to 55% in Kampala.

Most participants had disclosed their intention to use oral PrEP or the ring during the study to female family members (87.0%), friends (79.4%), and primary partners (60.3%), whereas only 23.9% of participants told male family members about intention to use the products (Table 3). When asked prior to initiation, how much they thought they would like to use each of the products for HIV prevention, 74.1% said they would like or like very much using the ring and 77.7% said they would like or like very much using oral PrEP. Baseline preference was similar for using the ring (38.1%) or oral PrEP (40.5%), with 19% of participants stating they preferred both products equally and only four (1.6%) stated they liked neither.

**Table 3:** Disclosure and preference for HIV prevention.

Characteristic	SA ‐ Cape Town	SA–Johannesburg	Uganda–Kampala	Zimbabwe–Harare	All Sites
**Participants with Enrollment ACASI**	60	67	60	60	247
**Disclosed to primary sex partner about intention to use oral PrEP or Ring**	36(60.0%)	47(70.1%)	31(51.7%)	35(58.3%)	149(60.3%)
**Disclosed to any female family member(s) about intention to use oral PrEP or Ring**	55(91.7%)	59(88.1%)	46(76.7%)	55(91.7%)	215(87.0%)
**Disclosed to any male family member(s) about intention to use oral PrEP or Ring**	18(30.0%)	20(29.9%)	9(15.0%)	12(20.0%)	59(23.9%)
**Disclosed to friend(s) about intention to use oral PrEP or Ring**	56(93.3%)	54(80.6%)	46(76.7%)	40(66.7%)	196(79.4%)
**Prefers to use the Ring or oral PrEP for HIV prevention**					
Ring	21(35.0%)	33(49.3%)	21(35.0%)	19(31.7%)	94(38.1%)
Oral PrEP	27(45.0%)	24(35.8%)	16(26.7%)	33(55.0%)	100(40.5%)
Either product equally	11(18.3%)	8(11.9%)	23(38.3%)	5(8.3%)	47(19.0%)
Neither product	1(1.7%)	1(1.5%)	0(0%)	2(3.3%)	4(1.6%)
Skipped by participant	0(0%)	1(1.5%)	0(0%)	1(1.7%)	2(0.8%)
**Pre-use opinion of using the Ring for HIV prevention**					
Dislike very much	4(6.7%)	0(0%)	3(5.0%)	12(20.0%)	19(7.7%)
Dislike	1(1.7%)	5(7.5%)	0(0%)	6(10.0%)	12(4.9%)
Neither like nor dislike	11(18.3%)	12(17.9%)	4(6.7%)	3(5.0%)	30(12.1%)
Like	27(45.0%)	25(37.3%)	21(35.0%)	30(50.0%)	103(41.7%)
Like very much	17(28.3%)	24(35.8%)	32(53.3%)	7(11.7%)	80(32.4%)
Skipped by participant	0 (0%)	1(1.5%)	0(0%)	2(3.3%)	3(1.2%)
**Pre-use opinion of using oral PrEP for HIV prevention**					
Dislike very much	3(5.0%)	2(3.0%)	1(1.7%)	2(3.3%)	8(3.2%)
Dislike	4(6.7%)	4(6.0%)	2(3.3%)	2(3.3%)	12(4.9%)
Neither like nor dislike	11(18.3%)	15(22.4%)	5(8.3%)	1(1.7%)	32(13.0%)
Like	19(31.7%)	32(47.8%)	23(38.3%)	35(58.3%)	109(44.1%)
Like very much	23(38.3%)	13(19.4%)	29(48.3%)	18(30.0%)	83(33.6%)
Skipped by participant	0(0%)	1(1.5%)	0(0%)	2(3.3%)	3(1.2%)

### Characteristics associated with preference for oral PrEP or the ring

We modeled the associations between baseline demographic and behavioral characteristics and preference for oral PrEP, the ring, or both products equally, controlling for site and age (Table 4). Furthermore, associations for site (controlling for age) and age (controlling for site) were also modeled. None of the characteristics were significant, except site (P<0.001, adjusted for age). Compared to participants from Zimbabwe, participants from Johannesburg had twice the odds of preferring the ring versus oral PrEP (OR 2.20; 95% CI 1.01–4.80) and participants from Uganda had nine times the odds of being interested in both methods equally versus oral PrEP (OR 9.14; 95% CI: 2.92–28.60). Although not statistically significant after adjusting for multiple comparisons, those who reported being at least a little worried about HIV acquisition had lower odds of preferring the ring (OR 0.47; 95% CI 0.23–0.99) and those who reported forced sex from non-intimate partner in the last 6 months also had lower odds of preferring the ring (OR 0.18; 95% CI 0.04–0.88). Those who reported condom use with last sex had twice to the odds of preferring the ring, and twice the odds of preferring both the ring and oral PrEP equally (OR 2.04; 95% CI 1.03–4.02) and (OR 2.36; 95% CI 1.03–5.44) respectively. Preference for ring or for both products was similarly higher with higher education level (OR 3.17; 95% CI 0.83–12.11) and (OR 4.24; 95% CI 0.97–18.51), respectively, although this was not statistically significant.

**Table 4:** Preference for oral PrEP or ring by baseline characteristics: Results from multinomial logistic regression with “Prefer PrEP” as reference level.

Characteristic	Prefer PrEP n (%)/mean (IQR)	Prefer Ring n (%)/mean (IQR)	Both equally n (%)/mean (IQR)	Prefer Ring Adjusted Odds Ratio (95% CI)	Both Equally Adjusted Odds Ratio (95% CI)
**Marital status** ^**1**^					
Single	87 (41%)	81 (39%)	42 (20%)	REF	REF
Married/ cohabitating	12 (43%)	12 (43%)	4 (14%)	1.44 (0.53–3.93)	1.03 (0.25–4.22)
Separated/divorced	1 (33%)	1 (33%)	1 (33%)	1.93 (0.11–34.00)	7.76 (0.38–156.86)
**Age** ^**2**^					
16–17	41 (49%)	27 (33%)	15 (18%)	REF	REF
18–21	59 (37%)	67 (42%)	32 (20%)	1.60 (0.87–2.95)	1.36 (0.63–2.95)
**Highest level of school attended** ^**1**^					
Secondary	84 (46%)	71 (39%)	29 (16%)	REF	REF
Tertiary	3 (13%)	12 (52%)	8 (35%)	3.17 (0.83–12.11)	4.24 (0.97–18.51)
Primary	13 (41%)	9 (28%)	10 (31%)	0.56 (0.17–1.87)	0.60 (0.17–2.14)
**Earns own income** ^**1**^					
No	82 (43%)	71 (37%)	37 (19%)	REF	REF
Yes	18 (35%)	23 (45%)	10 (20%)	1.44 (0.63–3.25)	0.52 (0.18–1.48)
**Pregnancy history** ^**1**^					
Never pregnant	63 (43%)	54 (37%)	29 (20%)	REF	REF
Ever pregnant	37 (39%)	40 (42%)	18 (19%)	1.33 (0.66–2.69)	0.96 (0.40–2.33)
**Worry about HIV infection** ^**1**^					
Not at all worried	24 (31%)	37 (47%)	17 (22%)	REF	REF
At least a little worried	76 (47%)	57 (35%)	30 (18%)	0.47 (0.23–0.99)	1.51 (0.56–4.04)
**Age of sexual debut** ^**1**^	15.9 (15.0–17.0)	16.0 (15.0–17.0)	15.6 (15.0–16.0)	0.94 (0.72–1.23)	0.79 (0.57–1.09)
**Baseline contraceptive method** ^**1**^					
Implants	45 (42%)	46 (43%)	17 (16%)	REF	REF
IUD	10 (37%)	10 (37%)	7 (26%)	1.08 (0.40–2.95)	2.25 (0.67–7.57)
Injectable contraceptive	45 (42%)	38 (36%)	23 (22%)	0.84 (0.43–1.64)	1.97 (0.81–4.82)
**AUDIT-C (categorical)** ^**1**^					
Negative (< 3)	62 (43%)	56 (39%)	27 (19%)	REF	REF
Positive (≥3)	38 (40%)	38 (40%)	20 (21%)	1.12 (0.60–2.09)	1.60 (0.72–3.57)
**Participant has a regular place to store own things** ^**1**^					
Yes	85 (43%)	78 (39%)	37 (19%)	REF	REF
No	15 (37%)	16 (39%)	10 (24%)	1.40 (0.63–3.10)	1.87 (0.72–4.86)
**Current living situation** ^**1**^					
Lives with parents	68 (45%)	53 (35%)	30 (20%)	REF	REF
Lives alone	2 (22%)	5 (56%)	2 (22%)	2.97 (0.52–17.05)	1.15 (0.13–9.75)
Lives with non-family members	6 (32%)	10 (53%)	3 (16%)	2.10 (0.69–6.35)	1.14 (0.25–5.23)
Lives with non-parent family members	15 (38%)	17 (43%)	8 (20%)	1.37 (0.61–3.11)	0.85 (0.30–2.42)
**Currently has primary sex partner** ^**1**^					
No	13 (48%)	10 (37%)	4 (15%)	REF	REF
Yes	87 (41%)	84 (39%)	43 (20%)	1.15 (0.47–2.83)	1.49 (0.44–5.13)
**Difference in age between participant and partner** ^**1**^	5.0 (2.0–7.0)	4.6 (2.0–7.0)	4.6 (3.0–6.0)	0.97 (0.88–1.06)	0.96 (0.85–1.08)
**Duration of partnership with primary partner** ^**1**^					
Less than 12 months	33 (40%)	31 (37%)	19 (23%)	REF	REF
More than 12 months	54 (42%)	52 (40%)	24 (18%)	0.90 (0.47–1.73)	0.77 (0.35–1.71)
**Partner provides financial support** ^**1**^					
No	30 (36%)	35 (42%)	18 (22%)	REF	REF
Yes	57 (44%)	49 (37%)	25 (19%)	0.70 (0.35–1.40)	0.50 (0.20–1.21)
**HIV status of primary partner** ^**1**^					
HIV negative	60 (38%)	66 (42%)	30 (19%)	REF	REF
HIV positive or don’t know	27 (47%)	18 (31%)	13 (22%)	0.66 (0.33–1.35)	1.15 (0.49–2.70)
**Physical abuse from intimate partner in last 6 months** ^**1**^					
No	86 (42%)	81 (39%)	39 (19%)	REF	REF
Yes	14 (40%)	13 (37%)	8 (23%)	1.25 (0.52–3.01)	1.53 (0.53–4.48)
**Verbal abuse from intimate partner in last 6 months** ^**1**^					
No	74 (44%)	63 (38%)	30 (18%)	REF	REF
Yes	26 (35%)	31 (42%)	17 (23%)	1.53 (0.79–2.97)	1.96 (0.86–4.46)
**Forced sex from intimate partner in last 6 months** ^**1**^					
No	88 (41%)	84 (39%)	42 (20%)	REF	REF
Yes	12 (44%)	10 (37%)	5 (19%)	0.85 (0.33–2.22)	0.56 (0.16–1.92)
**Forced sex from non-intimate partner in last 6 months** ^**1**^					
No	91 (41%)	92 (41%)	41 (18%)	REF	REF
Yes	9 (53%)	2 (12%)	6 (35%)	0.18 (0.04–0.88)	0.95 (0.28–3.23)
**Vaginal sex in last 6 months** ^**1**^					
No	24 (59%)	10 (24%)	7 (17%)	REF	REF
Yes	75 (38%)	84 (42%)	40 (20%)	2.21 (0.96–5.10)	1.73 (0.63–4.71)
**Condom use in last sex act** ^**1**^					
Yes	51 (46%)	42 (38%)	19 (17%)	REF	REF
No	24 (28%)	41 (48%)	21 (24%)	2.04 (1.03–4.02)	2.36 (1.03–5.44)
**Anal sex, last 3 months** ^**1**^					
No	89 (40%)	88 (40%)	45 (20%)	REF	REF
Yes	11 (58%)	6 (32%)	2 (11%)	0.55 (0.19–1.60)	0.28 (0.06–1.40)
**Number of male sex partners in last 3 months** ^**1**^					
≤ 1	67 (44%)	59 (39%)	27 (18%)	REF	REF
2+	33 (38%)	35 (40%)	20 (23%)	1.07 (0.58–1.96)	1.12 (0.52–2.40)
**Transactional sex in last 6 months** ^**1**^					
No	71 (41%)	72 (41%)	32 (18%)	REF	REF
Yes	29 (44%)	22 (33%)	15 (23%)	0.63 (0.31–1.29)	0.61 (0.25–1.47)
**Disclosure to primary sex partner about intention to use PrEP or Ring** ^**1**^					
No	42 (49%)	27 (31%)	17 (20%)	REF	REF
Yes	53 (37%)	62 (43%)	28 (20%)	1.78 (0.95–3.32)	1.48 (0.68–3.21)
**STI diagnosed at baseline** ^**1**^					
No	71 (46%)	59 (38%)	26 (17%)	REF	REF
Yes	29 (34%)	35 (41%)	21 (25%)	1.40 (0.75–2.61)	2.04 (0.94–4.40)
**Site** ^**3**^					
Zimbabwe ‐ Harare	33 (58%)	19 (33%)	5 (9%)	REF	REF
SA ‐ Cape Town	27 (46%)	21 (36%)	11 (19%)	1.25 (0.56–2.82)	2.56 (0.79–8.32)
SA ‐ Johannesburg	24 (37%)	33 (51%)	8 (12%)	**2.20 (1.01–4.80)**	2.08 (0.60–7.21)
Uganda ‐ Kampala	16 (27%)	21 (35%)	23 (38%)	2.16 (0.91–5.14)	**9.14 (2.92–28.60**)

^1^ Adjusted for site and age

^2^ Adjusted for site

^3^ Adjusted for age

## Discussion

The vanguard REACH crossover trial of the ring and oral PrEP implemented at four sites in three countries in Southern and East Africa demonstrates the feasibility of enrolling sexually active AGYW in Africa, with and without parental consent. Written informed assent and parental/guardian consent was obtained from all minors aged 16–17, and those who turned 18 during the study subsequently signed the ICF. The REACH cohort was diverse geographically with 34% of minors (16–17) and with an average age of 18 years old, an age group which experienced high HIV incidence in sub-Saharan Africa and poor adherence to oral PrEP and ring in previous studies [9, 10].

Prevalence of STIs in this cohort was high with similarly high rates of STIs being reported in studies of AGYW in other SSA settings [24–28]. These high rates of STIs are concerning especially because some STIs increase the possibility of HIV acquisition and the resultant long-term health consequences especially undiagnosed or untreated [25]. This high STI prevalence was further compounded by high reported intimate partner violence, including reports of forced sex (10.9%), an important contributor to HIV acquisition among AGYW [29].

The median age of sexual debut was a year earlier, on average, than starting contraceptive. A gap in protection against unintended pregnancy- which may explain the 40% who reported to ever been pregnant at this young age. This unmet need for contraception coupled with the high HIV risk highlights once again the twin challenges faced by these young women in SSA. Therefore, multipurpose technology products currently in development could be ideal for this population.

Multiple HIV prevention trials among AGYW have shown that adherence to oral PrEP and the ring can be challenging for AGYW [9, 10, 30–33]. Our study was designed to provide an opportunity for participants to experience both products during the randomized periods and provide an opportunity for AGYW to choose their preferred method during a third period. Preference for oral PrEP or the ring at baseline was not significantly different and the only factor predicting preference was study site. Given that pre-initiation product preference can inform initial choice among PrEP-naive AGYW, our findings suggest that multiple biomedical prevention options may be appealing to this age group and could address their prevention needs [34]. Our findings also add to the growing literature showing that interest and acceptability for different prevention methods vary by geography and cultural context, and thus, a menu of options would be most beneficial to increase protection coverage [35–38]. Our results are similar to the monitoring pre-exposure prophylaxis for young adult women (MPYA) study, which also found no association between demographic or behavioral factors and preference for oral PrEP or the ring [39]. Notably in the MPYA study participants had used PrEP for up to 24 months. However, our findings contrast with the tablets, ring, injections as options (TRIO) study results in which women using long-acting reversible contraception (LARC) at baseline preferred a ring over injections or tablets. Yet, just like in our study, TRIO found striking geographical differences in baseline product preference [40].

Mental health conditions, especially depression, have been shown to negatively affect adherence to oral PrEP, as well as ART initiation and continuation [16]. Data from our study suggests that nearly a third of participants had depressive symptoms according to the CESD-10 and an additional two-thirds had scores suggestive of alcohol abuse, which has also been shown to negatively affect adherence [41]. These high scores on both depressive symptoms and alcohol abuse point to the need for integration of mental health care into HIV prevention services for AGYW. Integration of these services has the potential not only to improve mental health but also could improve persistence on PrEP. Over 40% of participants had some worries about HIV acquisition, with a quarter reporting being very worried. Yet, substantial variations were found, and in some sites such as Kampala, most were not worried at all. Clear awareness of the potential for HIV transmission has been linked to high oral PrEP adherence observed in studies of HIV serodifferent couples [42]; conversely, low risk perceptions have been associated with poor oral PrEP uptake and continuation [43, 44]. It is therefore important to reframe HIV prevention messaging and steer away from a risk-framed message that is stigmatizing and use more empowering messaging for HIV prevention around health optimization, which are more effective [45, 46].

Disclosure of study products use reduces PrEP stigma and opposition, thereby improving adherence [47]. Notably, the ASPIRE study showed that there is a need to go beyond disclosure to providing support to avert opposition to product use after disclosure, for example through counselling and support groups [48]. In REACH, most participants had disclosed their intention to use PrEP products to female family members and primary sex partners prior to initiation, but not to male family members. In the VOICE-C study, male partners of study participants reported that they were more likely to support product use by their partners if they knew more about study participation and product use [49], and voluntary ring removals were associated with male partner concerns in the ASPIRE study [33]. Even though these products are designed for women’s use, there is a need to use innovative strategies to support women in making decisions about whether to disclose to male partners and family members. More product disclosure has the potential to help AGYW gain partner support and increase acceptance of product use more broadly. This can be achieved through couple counseling, face-to-face interactions with clinicians and service providers, and community education, which could translate to more community acceptance and effective use of prevention products [50].

This work has several limitations. Firstly, this study assessed cross sectionally stated preferences of prevention products at baseline before they had experienced using them. Future analysis from REACH will report informed preferences. Secondly our findings can only be generalizable to a narrow age group of AGYW aged 16–21, and to only two PrEP products; relative preferences for prevention products could differ for other age groups and as prevention options expand. Furthermore, the small sample size limited our ability to detect significant associations between baseline characteristics and product preferences. Thirdly, oral PrEP was already available in study countries when the trial began, whereas the ring was still under review by the regulatory authorities. It is possible that AGYW were more familiar with oral PrEP than the ring and that some participants had actually used PrEP before the trial, although this was not measured. These differences may have impacted initial responses. Despite these limitations, we were able to recruit a large proportion of minors [accompanied with parental consent], who were included for the first time in a ring trial, which will provide important safety, adherence and preference data for oral PrEP and the ring.

In conclusion, baseline data for REACH indicates that our cohort had important biological and behavioral vulnerability to HIV and thus an ongoing need for effective HIV prevention strategies. Additionally, a significant proportion of the AGYW exhibited low HIV risk perception and exhibited characteristics that have been shown to affect HIV prevention product adherence, such as depressive symptoms, alcohol abuse and lack of disclosure to partners. The balanced preference between the ring and oral PrEP suggests that multiple biomedical prevention options may be appealing to this age group and could address their prevention needs. Therefore, the REACH study is poised to provide important safety and adherence data among AGYW paving way for rollout of multiple PrEP options for this population.

## Supporting information

S1 File Inclusivity in global research. (DOCX)
